# Efficacy and Safety of Fire Needle Therapy for Nodular Prurigo: A Quantitative Study

**DOI:** 10.1155/2019/8797056

**Published:** 2019-06-25

**Authors:** Yue Luo, Le Kuai, Ningjing Song, Xiaojie Ding, Xiaoying Sun, Ying Luo, Yi Ru, Seokgyeong Hong, Meng Xing, Mi Zhou, Bin Li, Xin Li

**Affiliations:** ^1^Department of Dermatology, Yueyang Hospital of Integrated Traditional Chinese and Western Medicine, Shanghai University of Traditional Chinese Medicine, Shanghai 200437, China; ^2^Institute of Dermatology, Shanghai Academy of Traditional Chinese Medicine, Shanghai 201203, China; ^3^Department of Dermatology, Tongren Hospital, Shanghai Jiao Tong University School of Medicine, Shanghai 200050, China; ^4^Department of Dermatology, Shaanxi Traditional Chinese Medicine Hospital, Xi'an 710003, China

## Abstract

In this quantitative study, we evaluated the effectiveness and safety of fire needle therapy for nodular prurigo. We systematically searched several databases, including EMBASE, PubMed, the Cochrane Library, the Web of Science, the China Network Knowledge Infrastructure, the Wanfang Data Knowledge Service Platform, and the China Science and Technology Journal Database, and retrieved randomized controlled trials comparing conventional therapies (control group) with fire needle therapy alone or in combination with conventional therapies. Revman 5.2 software was used to calculate risk ratios (RR) with 95% confidence intervals (CI). In total, 14 trials with 1176 participants were included. Our quantitative study showed that the effectiveness rate of fire needle therapy combined with conventional therapies was significantly higher than that of conventional therapies alone (fire needle + traditional Chinese medicine [TCM] vs. TCM: RR, 1.11; 95% CI, 1.04 to 1.18; fire needle + oral thalidomide + topical glucocorticoid [TGC] vs. thalidomide + TGC: RR, 1.41; 95% CI, 1.17 to 1.70; fire needle + TGC vs. TGC only: RR, 1.18; 95% CI, 1.07 to 1.31). Similar results were obtained for the Symptom Score Reducing Index (fire needle + TCM vs. TCM: mean difference [MD], −3.39; 95% CI: −5.39 to −1.39), visual analog scale scores for itching severity (fire needle vs. halometasone cream: MD, −0.93; 95% CI, −1.29 to −0.58; fire needle + TCM vs. TCM: MD, −1.18; 95% CI, −1.78 to −0.58), and Dermatology Life Quality Index (fire needle vs. halometasone cream: MD, −3.03; 95% CI, −3.43 to −2.63; fire needle + TCM vs. TCM: MD, −2.53; 95% CI, −3.12 to −1.94). Adverse event and recurrence rates were comparable between groups. Thus, fire needle therapy alone or combined with conventional treatments may be effective for nodular prurigo, without any additional side effects.

## 1. Introduction

Nodular prurigo is a chronic inflammatory skin disease primarily characterized by severe itching papules and nodules. It often occurs in the limbs, particularly on the side of leg extension. The cause is unclear and the disease course is prolonged, which negatively affects the physical and mental health of patients. Current researches have suggested different causes. While one study stated that neurological factors such as substance P, calcitonin gene-related peptide, and interleukin-31 may play a mediating role in the pathogenesis of nodular prurigo, another implicated immunity-related factors and showed that helper T lymphocytes are decreased in patients with nodular pruritus, which suggests abnormal cellular immunity. Mental and psychological factors are also believed to play a role. Patients with nodular pruritus generally exhibit varying degrees of anxiety and depression and sensitive and introverted personalities [1, 2]. Current advanced therapies for this condition include topical steroids, capsaicin, calcineurin inhibitors, ultraviolet therapy, gabapentinoids, *μ*-opioid receptor antagonists, antidepressants, and immunosuppressants [3]. Treatments for this condition involve oral drugs such as antihistaminics or sedatives and topical drugs such as hormonal ointments; however, their curative effects are not significant. Intradermal injection of corticosteroids, liquid nitrogen freezing, and laser ablation can eliminate nodules and show therapeutic effects. However, these methods are not suitable for patients with a large number of lesions, which are likely to relapse [4, 5]. Fire needle therapy is a traditional treatment method considered to eliminate spirits and reduce swelling. The fire needle stimulates the meridians, dredges the meridians and collaterals, and accelerates the flow of Qi and blood, thus dissipating the nodules. In addition, it increases the nutrition around the lesion and promotes tissue regeneration, resulting in natural wound healing. From the perspective of modern medicine, the heat provided by fire needles promotes microcirculation in the lesion area through the regulation of cutaneous nerves, which is beneficial for the absorption of inflammation and metabolites. Furthermore, the high temperature of fire needles directly kills the microorganisms in the nodules and achieves anti-inflammatory effects [6]. In a previous study, it was speculated that hot fire needles can burn or even carbonize the nerve fibers in the skin lesions, slow down and block nerve conduction, and, consequently, alleviate pruritus [7]. This can be attributed to the increased density of SP-positive nerve fibers assumed to contribute to the induction and maintenance of pruritus, which has been demonstrated in patients with nodular prurigo [8, 9].

The aim of the present quantitative study was to gather evidence regarding the safety and efficacy of fire needle therapy for nodular prurigo in order to facilitate the clinical application of this treatment method.

## 2. Materials and Methods

This study has been registered with PROSPERO (CRD42019128168). It was performed according to the Cochrane Handbook for Systematic Reviews of Interventions [10] and is presented in accordance with the Preferred Reporting Items for Systematic Reviews and Meta-Analyses (PRISMA) guidelines (see Table S1 in the Supplementary Material).

### 2.1. Inclusion Criteria

Using the search terms “nodular prurigo” and “fire needle”, three reviewers (Yue Luo, Le Kuai, and Ying Luo) searched the following databases for relevant randomized controlled trials published from inception to February 2019: EMBASE, PubMed, the Cochrane Library, the Web of Science, the China Network Knowledge Infrastructure (CNKI), the Wanfang Data Knowledge Service Platform, and the China Science and Technology Journal Database (CQVIP). A total of 153 articles were retrieved, all of which were published in the Chinese databases.

### 2.2. Study Selection

We screened the titles, abstracts, and full texts of the 153 articles in order to identify those that met the following inclusion criteria: randomized controlled trials (RCTs) and observational studies, regardless of the use of blinding; inclusion of patients diagnosed with nodular prurigo, regardless of the age, sex, and ethnicity; use of fire needle therapy alone or in combination with conventional therapies; inclusion of control groups receiving any conventional therapy, without fire needle therapy, for nodular prurigo; and reporting of data regarding therapeutic efficacy and safety. The exclusion criteria were as follows: studies other than RCTs; theoretical explorations, case reports, reviews, and animal studies; and use of fire needle therapy in combination with a conventional therapy that was not used for the control group.

### 2.3. Data Extraction

Four investigators (Xin Li, Bin Li, Mi Zhou, and Xiaojie Ding) independently selected relevant studies after reading the titles and abstracts and further assessed the full texts of the selected studies. Another two researchers (Yue Luo; Le Kuai) completed the self-designed data extraction form, which included general information (i.e., the first author, year, objective, and study design), participant characteristics (i.e., disease duration, average age, and sample size), interventions, course of treatments, main outcomes, follow-up periods, AEs, and recurrence rates (RERs).

### 2.4. Methodological Quality Assessment

Four reviewers (Ying Luo, Meng Xing, Yi Ru, and Xijing Hong) independently assessed the risk of bias in the included studies. The evaluated parameters were as follows: random sequence generation, allocation concealment, blinding of participants and personnel, blinding of the outcome assessment, incomplete outcome data, selective reporting, and other biases. The results were subsequently classified into low risk, high risk, and unclear risk. The results were cross-checked by two investigators (Ningjing Song, Xiaoying Sun), and any disagreement was settled by a discussion between them.

### 2.5. Data Analyses

Review Manager (RevMan software, version 5.2, Cochrane Collaboration) [11] was used to identify differences in the main outcomes between the experimental (fire needle) and control groups. For dichotomous data, risk ratios (RR) with 95% confidence intervals (CI) were calculated. For continuous data, mean differences (MDs) and standard mean differences (SMDs) with 95% CIs were calculated. The degree of heterogeneity between studies was determined using the I^2^ statistic. A fixed model was applied when there was no significant heterogeneity (I^2^ < 50%); otherwise, a random effects model was considered suitable. A P-value of <0.05 was considered statistically significant.

### 2.6. Outcomes

The primary outcome was the total effectiveness rate for the duration of treatment. First, the effectiveness of treatment was divided into the following four categories: curative, defined by the resolution of itching and the skin lesions; markedly effective, defined by the partial resolution of or a reduction in itching, with resolution of >60% skin lesions; effective, defined by the partial resolution of or a reduction in itching, with resolution of >30% skin lesions; and ineffective, defined by the resolution of <30% skin lesions, with severe itching. Subsequently, the total effectiveness rate was calculated using the following formula: total effectiveness rate = (number of patients with curative treatment + number of patients with markedly effective treatment + number of patients with effective treatment)/total number of patients × 100%.

Secondary outcomes included the Symptom Score Reducing Index (SSRI), visual analog scale (VAS) score for itching severity, Dermatology Life Quality Index (DLQI) score, RER, and AEs.

## 3. Results

### 3.1. Selection and Characteristics of Studies

**Figure 1:**
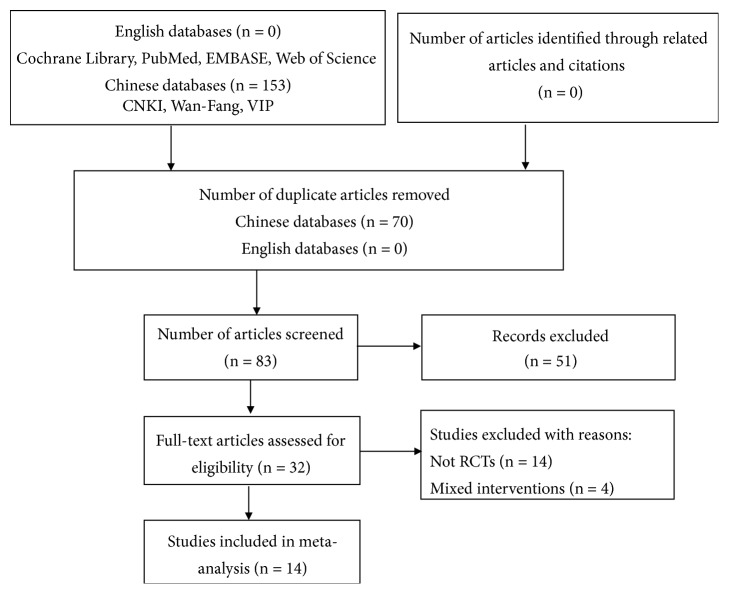
Study selection process for a quantitative study on the safety and efficacy of fire needle therapy for nodular prurigo.

From the 153 initially retrieved studies, 121 duplicate articles, theoretical explorations, case reports, and reviews were excluded after abstract and full text reviews. Another 14 non-RCTs and four studies with mixed interventions were eliminated. Finally, 14 RCTs [12–25] met the inclusion criteria and were included in our systematic review (Figure 1). All included studies were performed in China between 2014 and 2019, with two of them being unpublished master's theses [17, 19].

The characteristics of the included trials are listed in Table 1. A total of 1176 patients were included. From these, 627 and 549 patients belonged to the experimental and control groups, respectively. All patients were diagnosed with nodular prurigo according to clinical dermatology criteria in China [26]. Six RCTs compared fire needle therapy alone with conventional therapies, namely, halometasone cream [14, 19, 24], traditional Chinese medicine (TCM) [12, 22], and liquid nitrogen freezing [15], while 10 involved fire needle therapy combined with conventional therapies, namely, oral TCM [12, 13, 16, 17, 25], oral thalidomide + TGC [18, 23], and TGC alone [14, 20]. Two of the articles [12, 14] included two experimental groups (fire needle therapy alone and in combination) and one control group. Four studies [15, 19, 20, 24] reported recurrences, although only two of them [19, 20] calculated RERs. Seven studies [12, 15, 17–19, 23, 25] reported AEs. For evaluation of the efficacy, we combined healing, significantly effective, and effective outcomes into one positive category, while invalid was considered a negative category. These data were extracted as a dichotomous outcome. Two studies reported DLQI scores [17, 19], three reported VAS scores for itching [14, 19, 24], and five reported SSRI scores [12, 17, 19, 24, 25].

### 3.2. Risk of Bias

**Figure 2:**
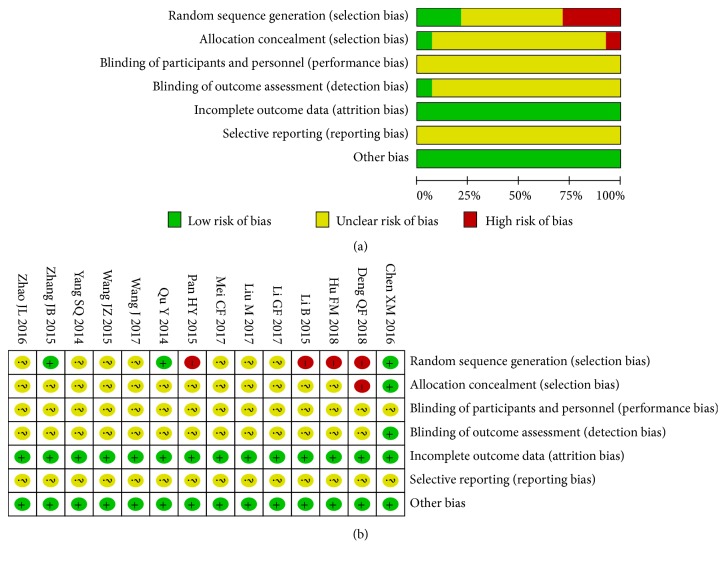
Risk of bias in the studies included in a quantitative study on the safety and efficacy of fire needle therapy for nodular prurigo. (a) Risk of bias graph. (b) Risk of bias summary.

The methodological quality was found to be poor for most of the included trials.  Figure 2 presents the risk of bias in all 14 studies. Although all studies were randomized trials, only six [12, 14, 15, 17, 19, 24, 25] documented their random sequence generation methods. The risk of bias in one study was high because grouping was performed according to the treatment method [24]. Only one trial [19] reported allocation concealment by use of opaque envelopes and described the method of blinding with regard to the outcome assessment. The other 13 studies did not document allocation concealment or blinding of participants, key study personnel, and outcome assessments. Complete outcomes were reported by all studies. Because the protocols of all 14 studies were not accessible, the selective reporting bias was considered unclear. The baseline characteristics (sex, age, disease severity, etc.) of participants in different treatment groups were found to be comparable.

### 3.3. Primary Outcomes

#### 3.3.1. Efficacy

**Figure 3:**
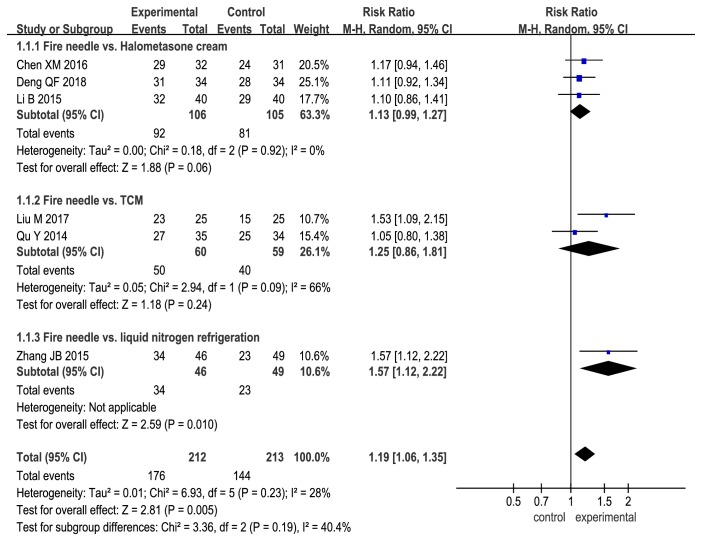
Forest plot comparing effectiveness rates between fire needle and control groups in a quantitative study on the safety and efficacy of fire needle therapy for nodular prurigo.

**Figure 4:**
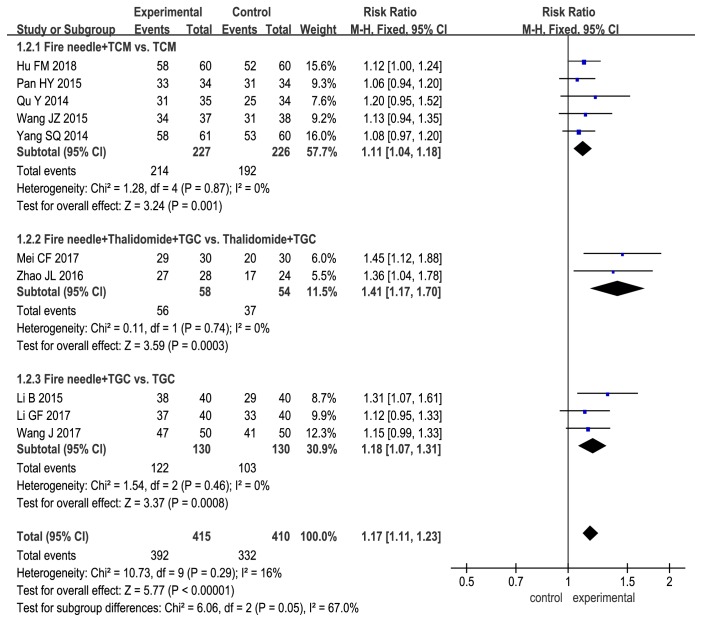
Forest plot comparing effectiveness rates between control treatments and fire needle therapy combined with other treatments in a quantitative study on the safety and efficacy of fire needle therapy for nodular prurigo.

Subgroup analysis demonstrated that the total effectiveness rate for fire needle therapy alone was significantly higher than that for physiotherapy (fire needle vs. liquid nitrogen freezing: RR, 1.57; 95% CI, 1.12 to 2.22; P = 0.01). However, the total effectiveness rate for fire needle therapy alone was comparable with those for topical halometasone cream and oral TCM (fire needle vs. halometasone: RR, 1.13; 95% CI, 0.99 to 1.27; P = 0.06; fire needle vs. TCM: RR, 1.25; 95% CI, 0.86 to 1.81; P = 0.24; Figure 3). When fire needle therapy was combined with conventional therapies, namely oral TCM, oral thalidomide + TGC, and TGC, the total effectiveness rate was significantly improved (fire needle + TCM vs. TCM: RR, 1.11; 95% CI, 1.04 to 1.18; P = 0.001; fire needle + thalidomide + TGC vs. thalidomide + TGC: RR, 1.41; 95% CI, 1.17 to 1.70; P = 0.0003; fire needle + TGC vs. TGC: RR, 1.18; 95% CI, 1.07 to 1.31; P = 0.0008; Figure 4).

### 3.4. Secondary Outcomes

#### 3.4.1. SSRI

**Figure 5:**
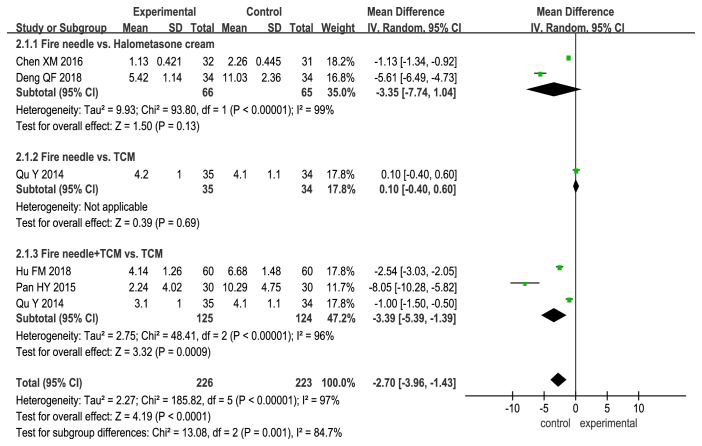
Forest plot comparing Symptom Score Reducing Index (SSRI) scores between fire needle and control groups in a quantitative study on the safety and efficacy of fire needle therapy for nodular prurigo.

We used SSRI to assess the curative effects of the evaluated therapies. The trends for the SSRI scores were consistent with those for the total effectiveness rate. We found that SSRI scores were significantly higher only when fire needle therapy was combined with conventional therapies (fire needle alone vs. halometasone cream: MD, −3.35; 95% CI, −7.74 to 1.04; P = 0.13; fire needle alone vs. TCM: MD, 0.1; 95% CI, −0.40 to 0.60; P = 0.69; fire needle + TCM vs. TCM: MD, −3.39; 95% CI, −5.39 to −1.39; P = 0.0009; Figure 5).

### 3.5. VAS Score for Itching Severity

**Figure 6:**
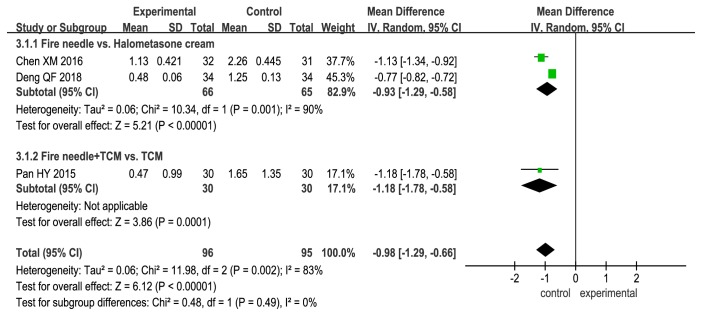
Forest plot comparing visual analog scale (VAS) scores for itching severity between fire needle and control groups in a quantitative study on the safety and efficacy of fire needle therapy for nodular prurigo.

VAS was used to evaluate the severity of itching in the different treatment groups, and the scores were found to be significantly lower for the experimental groups than for the control groups (fire needle alone vs. halometasone cream: MD, −0.93; 95% CI, −1.29 to −0.58; P < 0.00001; fire needle + TCM vs. TCM: MD, −1.18; 95% CI, −1.78 to −0.58; P = 0.0001; Figure 6).

### 3.6. DLQI Scores

**Figure 7:**
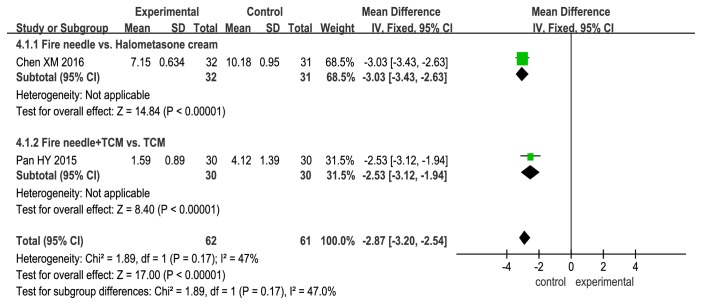
Forest plot comparing Dermatology Life Quality Index (DLQI) scores between fire needle and control groups in a quantitative study on the safety and efficacy of fire needle therapy for nodular prurigo.

DLQI scores for the experimental treatments were lower than those for the control treatments, thus indicating a better quality of life for the patients in the experimental groups (fire needle alone vs. halometasone cream: MD, −3.03; 95% CI, −3.43 to −2.63; P < 0.00001; fire needle + TCM vs. TCM: MD, −2.53; 95% CI, −3.12 to −1.94; P < 0.00001; Figure 7).

### 3.7. RER

**Figure 8:**
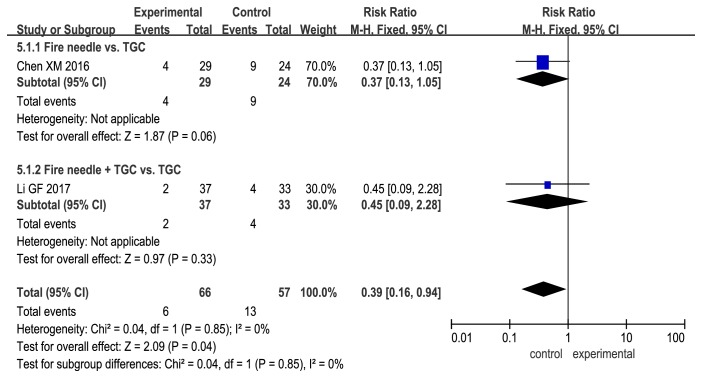
Forest plot comparing recurrence rate rates between fire needle and control groups in a quantitative study on the safety and efficacy of fire needle therapy for nodular prurigo.

Figure 8 shows that there was no significant difference in RER between the experimental and control groups (fire needle alone vs. TGC: RR, 0.37; 95% CI, 0.13 to 1.05; P = 0.06; fire needle + TGC vs. TGC: RR, 0.45; 95% CI, 0.09 to 2.28; Figure 8).

### 3.8. AEs

**Figure 9:**
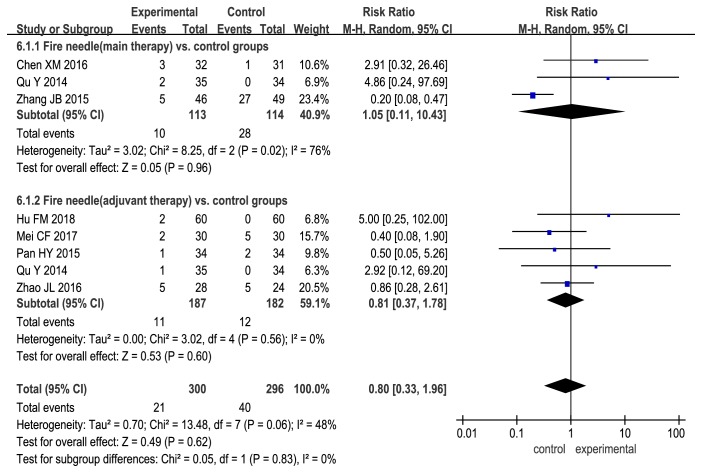
Forest plot comparing adverse events between fire needle and control groups in a quantitative study on the safety and efficacy of fire needle therapy for nodular prurigo.

The AE rate for fire needle therapy alone or in combination with other treatments was comparable to that for the control treatments (fire needle alone vs. control treatment: RR, 1.05; 95% CI, 0.11 to 10.43; fire needle in combination vs. control treatments: RR, 0.81; 95%CI, 0.37 to 1.78; Figure 9).

## 4. Discussion

Although the quality of the 14 RCTs included in the present study was not satisfactory, we could demonstrate, to a limited extent, the safety and efficacy of fire needle therapy for nodular prurigo. We found that the total effectiveness rate for fire needle therapy alone was significantly higher than that for liquid nitrogen freezing; however, the evidence was insufficient because this comparison was made in only one study [15]. The curative effect of modern medicine for nodular prurigo is well known, and our study also showed that the total effective rate for fire needle therapy alone was not significantly different from the rates for topical halometasone cream and oral TCM, which produces therapeutic effects by adjusting the balance of human Qi and blood as a whole.

However, it is interesting to note that the total effectiveness rate significantly increased when fire needle therapy was combined with conventional therapies, namely oral TCM, oral thalidomide + TGC, and TGC alone. SSRI scores showed a similar trend, with a significant improvement only when fire needle therapy was combined with conventional therapies. These findings suggest that adjuvant fire needle therapy in the treatment of nodular prurigo has beneficial effects. There could be several reasons for this important result. First, halometasone is a commonly used, highly potent TGC that inhibits inflammation, epidermal hyperplasia, and allergic reactions; constricts blood vessels; and relieves pruritus [27]. Moreover, the fire needles destroy the local skin barrier and increase the absorption of topically applied drugs [28]. Second, fire needle therapy is also a type of TCM, so its use in combination with oral TCM enhanced the therapeutic effects. Third, the therapeutic effects of the fire needle itself are combined with the curative effects of the conventional therapy.

In terms of the severity of itching, nodular prurigo is considered the worst condition among the different types of chronic pruritus [29]. It results in a decline in the quality of life, sleep disorders, and mental illness [29]. The treatment of nodular prurigo should be guided by two objectives: to minimize the itching and to resolve the lesions. In comparison with conventional therapies, fire needle therapy alone or in combination with conventional therapies could significantly alleviate itching, as assessed by VAS, and improve DLQI scores in patients with skin lesions in the present review. However, AEs and recurrences, which were comparable between the control and experimental groups, cannot be ignored. Nevertheless, our findings suggest that fire needle therapy does not increase the occurrence of AEs and recurrences. The included studies [8, 11, 13–15, 21] showed that the side effects in the fire needle group could disappear within a short period of time after the treatment of symptoms or without any special treatment. However, despite the enhanced effectiveness of combination fire needle therapies, the incidence of AEs was not different from that after conventional therapies.

This study has some limitations. First, the sample size was not large enough to draw reliable conclusions. Second, the quality of the included trials was not very high. Of the 14 trials, only three [12, 15, 19] reported specific randomization methods, while none involved blinding of the researchers, participants, and statisticians. Third, the number of events was very small [several subgroup analyses were included in only one study (Figures 3, 5, 6, 7, and 8)], and this may have influenced the results and their interpretation.

## 5. Conclusions

In conclusion, fire needle therapy combined with conventional treatments may be more effective than conventional therapies for nodular prurigo, with an AE rate similar to that for conventional treatments. However, our study did not find strong evidence supporting improved effectiveness when compared with conventional therapies. A large number of high-quality RCTs with low bias risks and adequate sample sizes are required to confirm the results of this quantitative study.

## Figures and Tables

**(a):** 

Study	Location	Baseline data comparable	Disease duration (average)		Average age (years)		Course of treatment (weeks)	Sample Size		FUP (months)	Adverse events	
			E	C	E	C		E	C		E	C
Qu Y, 2014	China	Yes	10.7 (6.1) y	10.7 (6.1) y	54.6 (16.5)	54.6 (16.5)	8	35	34	NR	Pain (2)	0

Yang SQ, 2014	China	Yes	NR	NR	NR	NR	4	61	60	NR	NR	NR

Li B, 2015	China	Yes	2 y	2 y	40	40	4	40	40	NR	Erythema and swelling (5)	0

Zhang JB, 2015	China	Yes	5.63 (3.22) y	5.78(3.12) y	23.42(4.51)	22.9(5.12)	4	49	46	6	Pain (5)	Blister (27)

Wang JZ, 2015	China	Yes	6.78 (3.6) y	6(3.29) y	32.91(6.91)	31.09(5,21)	4	37	38	NR	NR	NR

Pan HY, 2015	China	Yes	6 (2.85) m	5.91 (2.64) m	34.94(5.79)	35.06(6.19)	8	34	34	NR	Dizziness (1)	Irregular menstruation (1), stomach ache (1)

Zhao JL, 2016	China	Yes	18 m	18 m	41	41	4	28	24	NR	Thirst (2), drowsiness (2), erythema and swelling (1)	Thirst (2), drowsiness (3)

Chen XM, 2016	China	Yes	3.62 (1.55) y	3.51 (1.95) y	31.22 (5.64)	31.42 (5.27)	4	32	31	3m	Pain (2), pruritus with skin bruises (1)	Dizziness (1)

Li GF, 2017	China	Yes	5.1 (2.2) y	5.2 (2) y	40.3 (2.3)	42.1 (2.0)	4	40	40	6m	NR	NR

Wang J, 2017	China	Yes	2–8 y	2–8 y	38	38	4	50	50	NR	NR	NR

Liu M, 2017	China	Yes	9.23 (2.19) m	9.56 (2.36) m	39.88 (3.36)	38.98 (3.69)	4	25	25	NR	NR	NR

Mei CF, 2017	China	Yes	20.67 (10.28) m	18.63 (9.43) m	42.1 (19.48)	43.3 (14.38)	8	30	30	NR	Dizziness (2)	Thirst (2), drowsiness (3)

Deng QF, 2018	China	Yes	4.57 (1.6) y	4.23 (1.56) y	38.41 (4.62)	37.56 (4.56)	4	34	34	NR	NR	NR

Hu FM, 2018	China	Yes	2 (2.3) y	2 (2.5) y	35 (8.8)	38 (8.2)	4	60	60	NR	Erythema and swelling (2)	0

E: experimental group (fire needle therapy alone or combined with conventional therapy), C: control group (only the conventional therapy used in the experimental group), NR: no report, FUP: follow-up period, T: true, RCT: randomized controlled trial, w: weeks, m: months, y: years, SSRI: Symptom Score Reducing Index, VAS: visual analog scale, DLQI: Dermatology Life Quality Index, RER: recurrence rate, and AEs: adverse events.

**(b):** 

Study	RCT	Recurrence		Interventions		Main outcomes
		E	C	E	C	
Qu Y, 2014	T	NR	NR	Fire needle/fire needle + Longmu decoction	Longmu decoction	Total effectiveness rate, SSRI, AEs

Yang SQ, 2014	T	NR	NR	Fire needle + Quanchong prescription	Quanchong prescription	Total effectiveness rate

Li B, 2015	T	NR	NR	Fire needle/fire needle + Halometasone cream	Halometasone cream	Total effectiveness rate

Zhang JB, 2015	T	2	3	Fire needle	Liquid nitrogen refrigeration	Total effectiveness rate, AEs

Wang JZ, 2015	T	NR	NR	Fire needle + modified Wendan Decoction	modified Wendan decoction	Total effectiveness rate

Pan HY, 2015	T	NR	NR	Fire needle + Quanchong decoction	Quanchong decoction	Total effectiveness rate SSRI, VAS, DLQI, AEs

Zhao JL, 2016	T	NR	NR	Fire needle + Thalidomide +mometasone furoate	Thalidomide + mometasone furoate	Total effectiveness rate

Chen XM, 2016	T	4	9	Fire needle	Halometasone cream	Total effectiveness rate, SSRI, VAS, DLQI, RER, AEs

Li GF, 2017	T	2	4	Fire needle + Halometasone cream	Halometasone cream	Total effectiveness rate, RER

Wang J, 2017	T	NR	NR	Fire needle + Triamcinolone acetonide injection	Triamcinolone acetonide injection	Total effective rate

Liu M, 2017	T	NR	NR	Fire needle	Compound Wendan decoction	Total effectiveness rate

Mei CF, 2017	T	NR	NR	Fire needle + Thalidomide	Thalidomide	Total effectiveness rate, AEs

Deng QF, 2018	T	NR	NR	Fire needle	Halometasone cream	Total effectiveness rate, SSRI, VAS

Hu FM, 2018	T	NR	NR	Fire needle + Quanchong decoction	Quanchong decoction	Total effectiveness rate, SSRI, AEs

E: experimental group (fire needle therapy alone or combined with conventional therapy), C: control group (only the conventional therapy used in the experimental group), NR: no report, FUP: follow-up period, T: true, RCT: randomized controlled trial, w: weeks, m: months, y: years, SSRI: Symptom Score Reducing Index, VAS: visual analog scale, DLQI: Dermatology Life Quality Index, RER: recurrence rate, and AEs: adverse events.

## Data Availability

The data used to support the findings of this study are included within the article and the supplementary information files.

## Supplementary Materials

Supplementary Materials Table S1: PRISMA checklist.
